# Colistin-resistant *Escherichia coli* carrying *mcr-1* in food, water, hand rinse, and healthy human gut in Bangladesh

**DOI:** 10.1186/s13099-020-0345-2

**Published:** 2020-01-27

**Authors:** Fatema-Tuz Johura, Jarin Tasnim, Indrajeet Barman, Sahitya Ranjan Biswas, Fatema Tuz Jubyda, Marzia Sultana, Christine Marie George, Andrew Camilli, Kimberley D. Seed, Niyaz Ahmed, Munirul Alam

**Affiliations:** 1 Infectious Diseases Division (IDD), International Centre for Diarrheal Disease Research (icddr,b), 68, Shaheed Tajuddin Ahmed Sharani, Dhaka, 1212 Bangladesh; 2 Johns Hopkins Bloomberg School of Public Health, Baltimore, USA; 3 Tufts University, Medford, USA; 4 University of California, Berkeley, USA

**Keywords:** Colistin, *mcr-1*, ESBL, Multi-drug resistant (MDR), Minimum inhibitory concentration (MIC), Horizontal transmission

## Abstract

**Background:**

One of the most significant public health concerns in today’s world is the persistent upsurge of infections caused by multidrug resistant bacteria. As a result, clinicians are being forced to intervene with either less effective backup drugs or ones with substantial side-effects. Colistin is a last resort antimicrobial agent for the treatment of infections caused by multi-drug resistant gram-negative bacteria.

**Methods:**

*Escherichia coli* (n = 65) isolated from street food (n = 20), hand rinse (n = 15), surface water (n = 10), and healthy human stool (n = 20) were tested for colistin resistance gene *mcr-1* and response to antimicrobial agents. Antimicrobial resistance genes and virulence genes were detected by employing polymerase chain reaction. DNA fingerprinting of the strains were determined by pulsed-field gel electrophoresis.

**Results:**

Screening of *E. coli* allowed us to confirm colistin resistance marker gene *mcr-*1 in 13 strains (street food, n = 4; hand rinse, n = 2; surface water, n = 4; and stool, n = 3); and two of these *E. coli* strains carrying *mcr*-1 harbored *bla*_TEM_ gene encoding extended spectrum beta lactamase. Antibiotic assay results revealed all 13 *E. coli* strains carrying *mcr*-1 to be multi-drug resistant (MDR), including to colistin. The minimum inhibitory concentration (MIC) for colistin ranged from 2 to 6 μg/ml. DNA sequencing confirmed homogeneity of the nucleotide sequence for *mcr-1*, but the *E. coli* strains were heterogenous, as confirmed by pulsed-field gel electrophoresis suggesting horizontal transmission of colistin resistance in Bangladesh.

**Conclusion:**

Widespread dissemination of *E. coli* strains carrying *mcr*-1 encoding resistance to colistin in the present study is alarming as this is the last resort drug for the treatment of infections caused by MDR gram-negative bacteria resistant to almost all drugs used commonly.

## Background

Antimicrobial resistance (AMR) is one of the major global public health concerns of this century, which has made the effective treatment of an ever-increasing array of infectious diseases very challenging. The overuse of antibiotics in health and agriculture has put ubiquitous microbes under consistent selective pressure. As a result, only the microbes resistant to multiple antibiotics are thriving in the environment [1]. Bacteria belonging to the family *Enterobacteriaceae* include important pathogens that are ubiquitous in nature. The ability of *Enterobacteriaceae* to acquire mobile genetic elements carrying antibiotic resistance through horizontal transfer of genes has enabled this group to be the most successful in acquisition of resistance to multiple antibiotics, including the most effective next generation carbapenems [2].

In recent years there has been a tremendous increase in the incidence of critical infections in which the etiological agent is MDR gram-negative bacteria, in particular cephalosporin- and carbapenem-resistant *Enterobacteriaceae*. The ability to produce extended spectrum β-lactamases (ESBL) allows bacteria to be resistant to most beta-lactam antibiotics, including cephalosporin [3], an important drug of choice for treating both gram-positive and gram-negative bacterial infections. Widespread resistance to newer generations of cephalosporins is attributed largely to the spread of CTX-M type extended-spectrum β-lactamases (ESBLs) in gram-negative bacteria, especially in *Escherichia coli.* As a result, the rapid global dissemination of the ESBL-producing *E. coli* is an emerging public-health concern [4]. For ESBL and Ampicillinase C (AmpC) producers, carbapenems are the drugs of choice [5]**.** But dissemination of plasmid-mediated carbapenemases such as *Klebsiella pneumoniae* Carbapenemase (KPC) and metallo-lactamases, e.g. VIM, NDM-1 and IMP in carbapenem resistant *Enterobacteriaceae* (CRE) [6] left no drug to control them except for the last resort antibiotics, like colistin [7]**.**

Colistin (also known as polymyxin E) is a cationic polypeptide antibiotic that interacts with the outer membrane of the gram-negative bacteria [8]. Despite colistin once being avoided due to its nephro- and neurotoxicity [9]**,** this drug has now become a last-resort antimicrobial agent for treating life-threatening infections caused by MDR gram-negative bacteria. However, in November 2015, the first plasmid borne colistin resistance gene *mcr-1*, encoding a phosphoethanolamine transferase, was detected in livestock and raw meat samples as well as humans in China [10]. A recent study in Bangladesh reported *mcr-1* carrying *E. coli* in an urban sludge sample collected from Dhaka city [11]. Although little is known about the use of colistin in the clinical management of infectious diseases, this antibiotic has been widely used in the poultry industries of Bangladesh [12]**.** A recent study has reported *E. coli* carrying *mcr-3* from poultry in Moymonsingh, Bangladesh [13]. This cross-sectional study reported here was carried out in Dhaka city with the aim to understand the distribution of colistin resistant *E. coli* in street food and drink, water, hand rinse samples of street food-drink vendors, and stool from healthy humans.

## Materials and method

### Sample collection

To investigate the occurrence of colistin resistant bacteria, a total of 65 samples comprising healthy human stool (n = 20), street food and drink [n = 20; mixed fruit juice (n = 3), sugarcane juice (n = 2), Velpuri (a widely liked food item; n = 3), sliced guava mixed with pickles (n = 1), sliced pineapple mixed with pickles (n = 3), Peas cooked with spices (n = 3), and sliced cucumber (n = 5)], hand rinse samples of street food handlers (n = 15), and surface water (n = 10) were collected randomly from Dhaka city during June 2018. Human stools for healthy individuals were collected aseptically in stool collection cups and placed immediately in Cary-Blair media and transported to laboratory maintaining cold-chain. The water samples were collected using sterile 500 mL dark Nalgene bottles (Nalgene Nunc International, St. Louis, Mo.) and food samples were collected in 120 mL Whirlpak bag (NASCO WHIRL-PAK®, USA). For collection of hand rinse samples, street food handlers were asked to insert and wash their hands in 800-ml Whirl–Pak bag (NASCO WHIRL-PAK®, USA) containing 250 ml of sterile phosphate buffered saline. All samples were transported to the icddr, b laboratory in an insulated cool box (with ice packs).

### Isolation of* Escherichia coli*

The isolation of *E. coli* from all samples were performed using MacConkey agar (BD Difco, USA). Stool and juice samples were directly inoculated on MacConkey agar, while food samples were homogenized and diluted in PBS before inoculation on agar plates. For hand rinse and surface water samples, water was filtered through 0.22 μm filter papers and then the membrane filters were placed on MacConkey agar plate. The inoculated plates were incubated overnight at 37 °C. Bright pink lactose-fermenting colonies were selected as presumptive *E. coli*, which were grown on eosin methylene blue (EMB) agar to examine for the production of green colonies with a metallic sheen, a characteristic of important diagnostic implication for the bacterium. One presumptive colony per sample was selected at random, and subjected to biochemical confirmation with API 20 E (BioMerieux, France).

### Detection of colistin resistance *mcr-1* gene

Bacterial DNA was obtained from all isolates by the boiling method [14]. All *E. coli* strains were examined for the presence of *mcr-1* gene by polymerase chain reaction (PCR) using primers as described elsewhere [10]. The primer sequences and corresponding annealing temperatures used in all PCR reactions in this study are listed in Table 1.

**Table 1:** PCR primers used in this study

Target gene	Primer	Sequence	Amplicon size (bp)	References
*mcr1*	CLR5F	CGGTCAGTCCGTTTGTTC	309	[10]
	CLR5R	CTTGGTCGGTCTGTA GGG		
LT	LT 450F	GGCGACAGATTATACCGTGC	450	[21]
	LT 450R	CGGTCTCTATATTCCCTGT		
*STp*	STp186F	TCTGTATTATCTTTCCCCTC	186	[22]
	STp 186R	ATAACATCCAGCACAGGC		
*STh*	STh5f	TCACCTTTCCCTCAGGAT	160	[23]
	STh6r	TACAAGCAGGATTACAACAC		
*pCVD432*	EAEC1	CTGGCGAAAGACTGTATCAT	630	[24]
	EAEC2	CAATGTATAGAAATCCGCTGTT		
*bla*_CTX-M-15_	CTX-M-15F	CACACGTGGAATTTAGGGACT	996	[25]
	CTX-M-15R	GCCGTCTAAGGCGATAAACA		
*bla*_CTX-M-2_	CTXM2F	CGGYGCTTAAACAGAGCGAG	891	[26]
	CTXM2R	CCATGAATAAGCAGCTGATTGCCC		
*bla*_CTX-M-8_	CTXM8F	ACGCTCAACACCGCGATC	490	[26]
	CTXM8R	CGTGGGTTCTCGGGGATAA		
*bla*_CTX-M-9_	CTXM9F	GATTGACCGTATTGGGAGTTT	947	[26]
	CTXM9R	CGGCTGGGTAAAATAGGTCA		
*bla*_TEM_	TEM-R	ACGCTCAGTGGAACGAAAAC	1150	[27]
	TEM-F	ATTCTTGAAGACGAAAGGGC		
*bla*_NDM1_	NDM-1F	GGTTTGGCGATCTGGTTTTC	621	[28]
	NDM-1R	CGGAATGGCTCATCACGATC		
*bla*_OXA-48_	OXA-F	GCGTGGTTAAGGATGAACAC	438	[29]
	OXA-R	CATCAAGTTCAACCCAACCG		
*bla*_CMY-2_	CMY-2-R	CCCGTTTTATGCACCCATGA	870	[30]
	CMY-2-F	TGGCCGTTGCCGTTATCTAC		
*mphA*	mphA-F	GTGAGGAGGAGCTTCGCGAG	403	[31]
	mphA-R	TGCCGCAGGACTCGGAGGTC		

### Sequencing of *mcr-1* gene

Amplified fragment of *mcr-1* was sequenced using an ABI PRISM Big Dye Terminator Cycle Sequencing Reaction kit (Applied Biosystems) on an ABI PRISM 310 automated sequencer (Applied Biosystems). The raw sequences were subjected to sequence analysis software (Chromas), the sequences were then searched for homology using Basic Local Alignment Search Tool (BLAST). The partial sequences of the gene were submitted to GenBank (Accession Numbers: MN337021, MN337022, MN337023, MN337024, and MN337025).

### Determination of minimum inhibitory concentration (MIC) of colistin

The *mcr-1* positive strains were tested for their response to colistin by measuring the MIC by E-test (BioMerieux). The results were interpreted according to European Committee on Antimicrobial Susceptibility Testing (EUCAST) breakpoints [15]. Although broth microdilution assay is recommended by EUCAST for determining MIC, several studies have found a congruous correlation between the E-test and reference techniques [16-18].

### Antimicrobial susceptibility tests

Colistin resistant strains were tested for their susceptibility to other antimicrobials following the disc diffusion method as described by Bauer et al. [19] using commercially available antibiotic discs. Eighteen commonly used antibiotics (Oxoid, UK) tested in this study include: ceftriaxone (CRO 30 µg), cephalothin (KF 30 µg), cefipime (FEP 30 µg), cefixime (CFM 5 µg), fosfomycin (FOS 50 µg), mecillinam ( MEL 25 µg), tetracycline (TE 30 µg), sulphamethoxazole-trimethoprium (SXT 25 µg), levofloxacin (LEV 5 µg), erythromycin (E 15 µg), azithromycin (AZM 15 µg), imipenem (IPM 10 µg), ampicillin (AMP 10 µg), nalidixic Acid (NA 30 µg), ciprofloxacin (CIP 5 µg), gentamicin (CN 10 µg), chloramphenicol (C 30 µg), and aztreonam (ATM 30 µg). The resistance or susceptibility profiles of the isolates were determined by measuring the inhibitory zone and comparing it with an interpretative chart to determine sensitivity to the antibiotics according to Clinical and Laboratory Standards Institute guideline [20]. *E. coli* ATCC 25922 was used as a positive control.

### Detection of virulence gene

Colistin resistant *E. coli* isolates were tested for the presence of genes for ETEC-specific heat-labile toxin (*lt*) and heat stable toxin (*STp, STh*), EAEC virulence plasmid pCVD432 by PCR.

### Detection of genes associated with ESBL carbapenem and macrolide resistance

The colistin resistant *E. coli* strains were targeted to detect ESBL genes (*bla*_CTX-M-15,_ *bla*_CTX-M-2-group_, *bla*_CTX-M-8-group_, *bla*_CTX-M-9-group,_ *bla*_TEM_), carbapenemase genes (*bla*_NDM-1_ *bla*_OXA -48,_* bla*_CMY-2_), and macrolide resistance gene (*mphA)* by PCR.

### Pulsed-field gel electrophoresis (PFGE)

*mcr*-1^+^ *E. coli* strains were analyzed using pulsed-field gel electrophoresis (PFGE) according to standard protocols [32]. The fingerprint patterns were typed according to banding similarity and dissimilarity, using the dice similarity coefficient and unweighted-pair group method employing average linkage (UPGMA) clustering, as recommended by the manufacturer. The results were graphically represented as dendrograms.

## Results and discussion

A total 65 *E. coli* were isolated by culture methods from street food (n = 20), hand rinse (n = 15), surface water (n = 10), and healthy human stool (n = 20). The genomic DNA of these strains were subjected to PCR using specific primers for the colistin resistance gene *mcr-1* [10]. Of the 65 *E. coli* strains tested, 13 (20%) (Street food; 4, hand rinse; 2, surface water; 4, and healthy human gut; 3) carried a 309 bp amplicon specific for the *mcr-*1 gene (Table 2). The 309 bp fragments of *mcr-*1 from five representative *E. coli* strains from healthy human stool (n = 2), water (n = 1), hand rinse (n = 1), and street food (n = 1) were subjected to nucleotide sequencing (Genbank Accession No. MN337021, MN337022, MN337023, MN337024, and MN337025). Although it was not possible in this study to cover the full-length sequence of the gene, BLAST homology search demonstrated the nucleotide sequences to be identical to that of the corresponding *mcr-1* portion reported earlier from *E. coli* strains (Genbank Accession No. KP347127, NG056412, CP015913, KY770023, and KY550358). These previously reported sequences were found in *E.coli* isolated from animal and water sources of China, Japan, USA, Brazil and South America. *E. coli* carrying *mcr-1* has been reported in recent years from urban sludge samples from Bangladesh [11]. The data in the present study show how rapidly the colistin resistant *E. coli* carrying *mcr-1* has been disseminating, as those were found from street foods, hand rinse samples of the street food vendors, and healthy human gut in Dhaka, a densely populated city of Bangladesh. Although the study was limited to Dhaka, the results may be indicative of the prevalence and spread of the colistin resistant *E. coli* throughout Bangladesh.

**Table 2:** MIC value and drug resistance pattern of colistin resistant *mcr-1* carrying *E. coli* (n = 13) strains

Source	No. of isolates	*mcr-1*	MIC of colistin (μg/ml)	Resistance pattern
Street drink (sugarcane juice)	1	+	4	KF, CFM, CRO, TE, SXT, LEV, E, AZM, AMP, NA, CN, CIP, ATM
Street drink (sugarcane juice)	1	+	4	KF, TE, SXT, LEV, E, AMP, NA, CIP.CN
Street drink (mixed fruit juice)	1	+	3	TE, SXT, E, AMP, CN
Street drink (mixed fruit juice)	1	+	6	KF, TE, SXT, E, AZM, AMP, CN, NA
Hand rinse	1	+	4	TE, SXT, E, AMP, NA
Hand rinse	1	+	2	KF, TE, SXT, LEV, E, AMP, NA, CIP, C
Surface water	1	+	3	TE, SXT, E, AMP, NA
Surface water	1	+	4	TE, SXT, AZM, E, AMP, NA
Surface water	1	+	4	KF, TE, SXT, LEV, E, AZM, AMP, NA, CN, CIP, C
Surface water	1	+	4	KF, TE, AMP,
Healthy human stool	2	+	4	KF, TE, SXT, LEV, E, AZM, AMP, NA, CIP, CN
Healthy human stool	1	+	3	KF, TE, SXT, E, AMP, CN

*CFM* cefixime, *KF* cephalothin, *CRO* ceftriaxone, *TE* tetracycline, *SXT* sulphamethoxazole/trimethoprium, *LEV* levofloxacin, *E* erythromycin, *AZM* azithromycin, *AMP* ampicillin, *NA* nalidixic acid, *CN* gentamicin, *CIP* ciprofloxacin, *ATM* aztreonam, *C* chloramphenicol

*Escherichia coli* strains carrying *mcr-1* were found to be resistant to colistin in the present study. The MIC value was determined to range from 2 to 6 μg/ml. Using the Kirby–Bauer disk diffusion assay, we showed that these colistin resistant *E. coli* strains were MDR with resistance varying from 3 to 13 different antibiotics. Among the 13 *mcr-1* carrying *E. coli* strains, 100% (13/13) were resistant to tetracycline and ampicillin, 92% (12/13) resistant to erythromycin and sulphamethoxazole-trimethoprium, 77% (10/13) resistant to nalidixic acid, 69% (9/13) resistant to cephalothin, 62% (8/13) resistant to gentamycin, 46% (6/13) resistant to levofloxacin, azithromycin and ciprofloxacin, 15% (2/13) resistant to chloramphenicol, and 8% (1/13) resistant to cefixime, ceftriaxone and aztreonam. All of the *mcr-1* carrying *E. coli* strains were sensitive to fosfomycin, mecillinam, imipenem, and cefipime. Only one strain was resistant to 3rd generation cephalosporins (cefixime and ceftriaxone). The widespread occurrence of colistin resistant MDR *E. coli* is alarming for a densely populated country like Bangladesh where morbidity and mortality due to infectious diseases are prevalent [12, 33]. Transmission control of colistin resistant bacteria warrant urgent attention, although further studies would be required to comprehend the transmission from community to clinical settings.

**Fig. 1:**
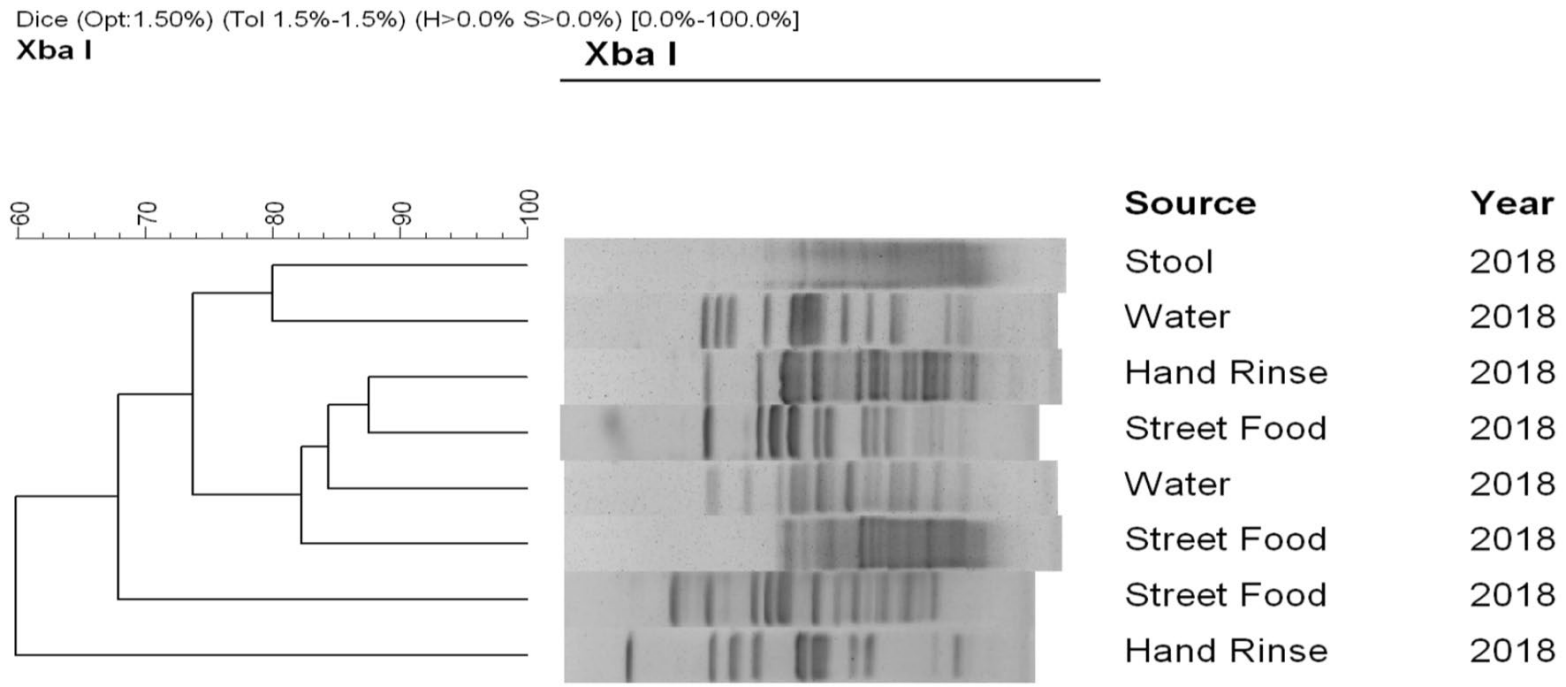
Dendrogram showing genomic relatedness of *mcr-1* carrying *E. coli* strains. Pulsed-field gel electrophoresis (PFGE) patterns of *Xba*I-digested genomic DNA of representative *mcr-*1 carrying *E. coli* strains isolated from healthy human gut, water, street food and hand rinse of street food vendors. The dendrogram was prepared by the BioNeumeric software (Applied Maths) using dice similarity coefficient and unweighted-pair group method employing average linkage of the PFGE images of the *E. coli* strains. The scale bar at the top (left) indicates similarity coefficient (%).

We tested all of the *mcr-1* positive *E. coli* strains for the presence of other important antimicrobial resistance marker genes. Of the 13 *mcr-1* carrying *E. coli* strains, only two isolated from street food and healthy human gut harbored the ESBL gene *bla*_*TEM*_. Also, eight *E. coli* strains isolated from street food (n = 3), surface water (n = 2) and healthy human gut (n = 3) had the macrolide resistance gene *mphA*, which reflects the higher percentage of erythromycin and azithromycin resistance within the isolates (Table 3). Although we have not tested the strains for plasmid carriage, ESBL and *mcr-1* genes can co-exist on the same plasmid and therefore could facilitate dissemination of colistin resistance [34]. The *E. coli* strains carrying *mcr-1* were heterogenous genetically, as confirmed by PFGE (Fig. 1), suggesting horizontal transmission of the antimicrobial resistance genes. None of the *mcr-1* carrying *E. coli* strains in the present study are believed to be pathogenic as they did not carry any of the virulence and related genes namely *LT, STp, STh* and *pcvd432* plasmid. Nonetheless, the occurrence of colistin resistant bacteria in the human gut was important as they could serve as a reservoir for future transfer to pathogenic strains.

**Table 3:** Virulence and resistance genes profile of *mcr-1* carrying *E. coli*

Source	No. of isolates	Virulence gene profile				Resistance gene profile								
		*LT*	*STp*	*STh*	*pcvd 432*	*bla*_OXA-48_	*bla*_CMY-2_	*bla*_NDM-1_	*bla*_TEM_	*bla*_CTX-M-15_	*bla*_CTX-M-2_	*bla*_CTX-M-8_	*bla*_CTX-M-9_	*mphA*
Street food (sugarcane juice)	1	−	−	−	−	−	−	−	+	−	−	−	−	+
Street food (sugarcane juice)	1	−	−	−	−	−	−	−	−	−	−	−	−	−
Street food (mixed fruit juice)	2	−	−	−	−	−	−	−	−	−	−	−	−	+
Hand rinse	2	−	−	−	−	−	−	−	−	−	−	−	−	−
Surface water	2	−	−	−	−	−	−	−	−	−	−	−	−	+
Surface water	2	−	−	−	−	−	−	−	−	−	−	−	−	−
Healthy human gut	1	−	−	−	−	−	−	−	+	−	−	−	−	+
Healthy human gut	2	−	−	−	−	−	−	−	−	−	−	−	−	+

## Conclusion

The usefulness of colistin, a last-resort antibiotic for the treatment of MDR gram-negative bacterial infections, is being compromised as shown by the recent identification of the mobile colistin resistance gene, *mcr-1* [10]. The *mcr-1* gene has spread to most continents, and has been detected in various bacterial isolates from animals, human and the environment, including *E. coli*, *Klebsiella pneumoniae*, *Enterobacter cloacae* and *Enterobacter aerogenes* [35]. In Bangladesh, colistin resistant *E. coli* carrying *mcr-1* was reported earlier from urban sludge samples [11]. Here in this study, colistin resistant *E. coli* carrying *mcr-1* gene was found from water, street food, hand rinse samples of street food vendors, and healthy human gut samples in Bangladesh. This is alarming and sheds light on the potential health risk that colistin resistant *E. coli* could pose to millions of people in Bangladesh as colistin is a last resort antibiotic for the treatment of MDR gram-negative bacterial infections.

## Data Availability

Sequence data that support the findings of this study have been deposited in GenBank with the primary accession codes MN337021, MN337022, MN337023, MN337024, and MN337025.
